# Purifying Drinking Water with Sun, Salt, and Limes

**DOI:** 10.1289/ehp.120-a305

**Published:** 2012-08-01

**Authors:** Adrian Burton

**Affiliations:** Adrian Burton is a biologist living in Spain who also writes regularly for *The Lancet Oncology*, *The Lancet Neurology*, and *Frontiers in Ecology and the Environment*.

Sun, salt, and lime juice may sound like ingredients for a vacation margarita, but recent research suggests they can also be used to help purify drinking water easily and cheaply—the type of solutions needed by millions of people in developing countries. Some 780 million people across the globe are still without reliable access to safe drinking water.^1^ Bringing safe water to these people will depend on making affordable, technically feasible solutions available.

The solar disinfection of drinking water, or SODIS, method is one such solution now being used by more than 5 million people in 24 African, Asian, and Latin American countries.^2^ Water poured into clear or blue polyethylene terephthalate water bottles (glass bottles also can be used) is exposed to sunlight for at least 6 hours, or up to 48 hours in cloudy weather. The heat and ultraviolet radiation of the sunlight kill bacteria and protozoan parasites and inactivate assorted viruses. This method is reported to significantly reduce the number of children falling ill to diarrheal diseases (some studies suggesting by up to 70%) and cholera (by approximately 86%).^3^ Although questions have been raised about the leaching of plasticizers and other hormonally active chemicals from heated plastic bottles, studies to date indicate the SODIS method does not impart an unusual burden of endocrine disruptors to drinking water.^3^

However, if the water placed in the bottles is very turbid, the method is rendered ineffective as soil particles in the water shield microorganisms from the disinfecting rays.^4^ Filtering the water before bottling it can solve this problem, but the additional equipment required may not be available.

New work suggests, however, that adding a tiny amount of salt to turbid water causes the suspended clay particles to flocculate and sink to the bottom of the bottle, leaving clear water that can be decanted and subjected to the SODIS method. Using distilled water, researchers prepared 1-liter suspensions of bentonite, kaolinite, and illite (typical clays of tropical regions) with turbidities of 50, 100, and 200 nephelometric turbidity units (NTU). Then they added salt at a range of concentrations to determine how much was needed to reduce turbidity below 30 NTU, the threshold at which SODIS functions.

Adding 1,250 mg salt (about a quarter teaspoon) per bottle brought all three 50-NTU suspensions to below this threshold within 1 hour. The more turbid solutions of bentonite, which flocculated more easily than the other clays, required less salt (1,000 mg/L) to achieve the same; the greater proximity of the clay particles to one another makes flocculation easier. The more turbid kaolinite and illite suspensions required the addition of a bentonite “jumpstarter” in order to be brought below the SODIS threshold.^4^ None of the treated samples in the study had salt concentrations below the taste threshold of 256 mg/L, but several had less residual salt than a typical sports drink.

**Figure:**
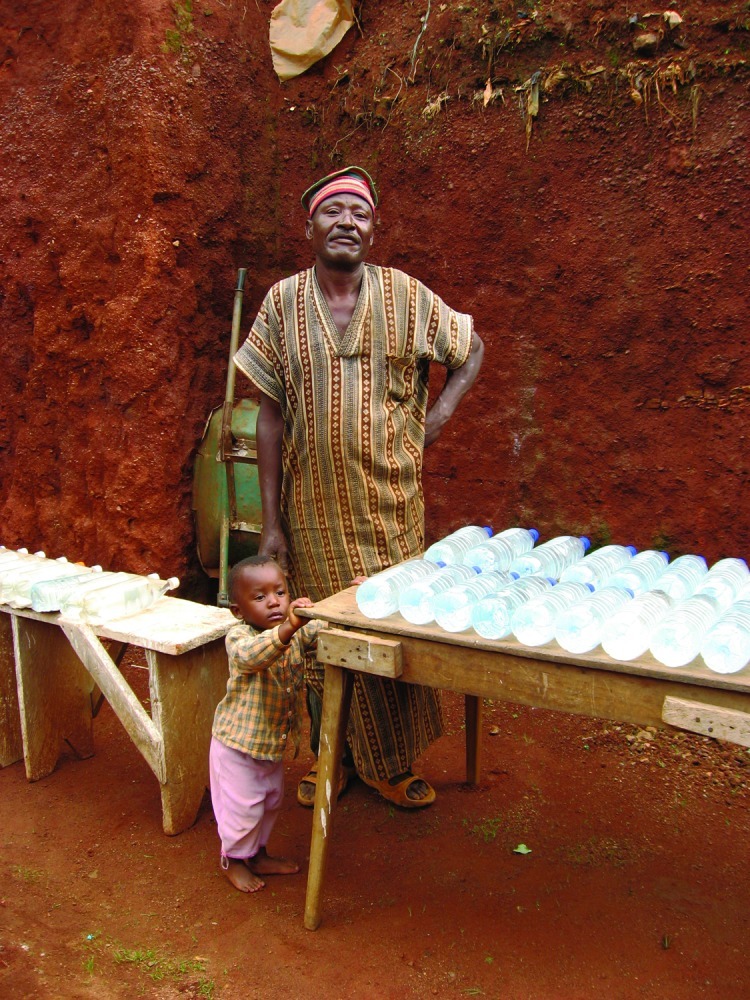
In the slums of Yaoundé, Cameroon, more than 50,000 people—including this grandfather and his grandchild—use the SODIS method daily to treat their drinking water. © Eawag/Sandec

According to research leader Joshua Pearce, an associate professor in the Department of Materials Science and Engineering at Michigan Technological University, if you can read the headlines of a newspaper placed under a bottle of water, you have reduced the turbidity to below the 30-NTU SODIS threshold. “This provides an easy-to-understand sign for people outside of a laboratory that water has been sufficiently cleared for the SODIS treatment to work,” he says.

In other recent research, lime juice has been shown to speed up the disinfecting power of the SODIS method.^5^ Limes contain large quantities of psoralens. These molecules form covalent crosslinks between DNA strands in the presence of sunlight, a reaction that prevents DNA replication.^6^ This team prepared 2-liter plastic bottles of tap water spiked with 30 mL lime juice (about half a lime’s worth) or 60 mL lime “slurry” produced by chopping, homogenizing, and centrifuging the entire fruit. They then added populations of either *Escherischia coli*, bacteriophage MS2 (a surrogate for many human viruses), or murine norovirus (MNV; a surrogate for human norovirus), and exposed the bottled waters to sunlight for up to 6 hours.

Both lime slurry and plain lime juice, combined with SODIS, reduced *E. coli* by roughly 1 million times in just 30 minutes, whereas SODIS on its own was about one-quarter as effective. Reductions in MS2 were measured over a 2.5-hour solar exposure, with viable virus particles reduced by about 10,000 and 100 times with the lime slurry and lime juice, respectively, compared with a 25-times reduction by SODIS alone. The MNV virus was only modestly affected by any combination of methods.^5^

“Since limes are available in many places where SODIS is practiced, lime juice could be a useful way to speed up the process,” says first author Alexander Harding, a medical student at the Johns Hopkins University School of Medicine. However, limes may not be available everywhere, and not all citrus fruits have such high psoralen concentrations, meaning limes might not be replaceable by other citrus fruits.

“These research findings are useful, potentially improving the effectiveness of the SODIS approach,” says Vincent Casey, technical support manager at the London offices of the nongovernmental organization WaterAid. But he points out there is no silver bullet solution that addresses all water quality challenges. Rather, a combination of approaches is required to ensure adequate water quality and disease prevention. For many communities, however, these new SODIS-associated techniques may be a step in the right direction.
